# Transient cerebral vasospasm and global amnesia following post-CT contrast^☆^

**DOI:** 10.1016/j.radcr.2024.04.079

**Published:** 2024-05-17

**Authors:** Naoki Kiyoshige, Keita Watanabe, Tomoaki Nishimura, Takaaki Ito, Kiyoshi Ishihara, Kei Yamada

**Affiliations:** a Department of Radiology, Kyoto Yamashiro General Medical Center, 1-27 Kizuekimae, Kizugawa, Kyoto, 036-8562, Japan; b Department of Radiology, Kyoto Prefectural University of Medicine, 465 Kajiimachi, Kamigyo-ku, Kyoto, 602-8556, Japan

**Keywords:** Contras material, Vasospasm, Transit amnesia

## Abstract

We present a unique case of transient global amnesia following intravenous administration of a non-ionic iodinated contrast agent for abdominal CT examination. Follow up MR imaging and MR angiography studies revealed hippocampal microinfarction and transient cerebral vasospasm. To our knowledge, this is the first reported case capturing arterial vasospasm following intravenous use of iodinated contrast. Medical professionals handling contrast agents should note the potential for these rare but serious adverse effects.

## Introduction

Contrast agents are routinely used in radiological examinations to enhance the visibility of organs, lesions, and blood vessels. While these agents are generally considered safe, they are not devoid of adverse effects. Common side effects range from mild allergic reactions to more severe instances of contrast-induced nephropathy [1]. In vascular imaging, arterial administration of contrast media is known to occasionally provoke vasospasm. However, upon reviewing the literature, we found no reported cases of cerebral arterial vasospasm following intravenous (IV) administration of contrast agents. This absence of documentation highlights the importance of clinical vigilance and the need for further reporting. In this context, we encountered a rare instance where transient global amnesia developed after contrast-enhanced CT for the evaluation of a splenic artery aneurysm.

## Case report

**Fig. 1:**
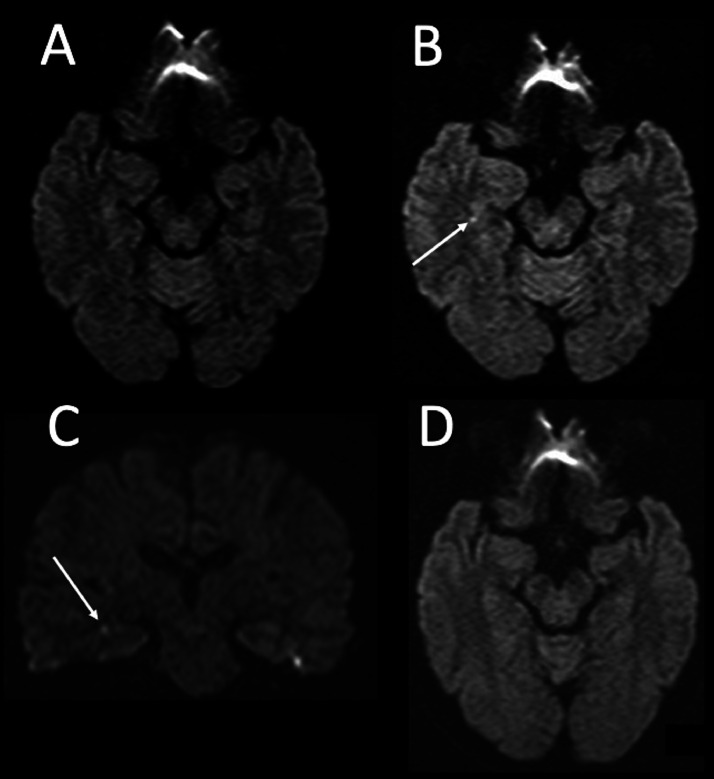
Time course of hippocampal microinfarction on diffusion weighted imaging. (A) Axial diffusion-weighted imaging (DWI) obtained 3 hours post-onset, showing no significant signal changes. (B, C) Axial and coronal DWI, respectively, obtained 2 days post-onset, both demonstrating a punctate high signal in the right hippocampus (arrow). (D) Axial DWI obtained 9 days after onset, showing disappearance of the previously observed abnormal signal in the right hippocampus.

**Fig. 2:**
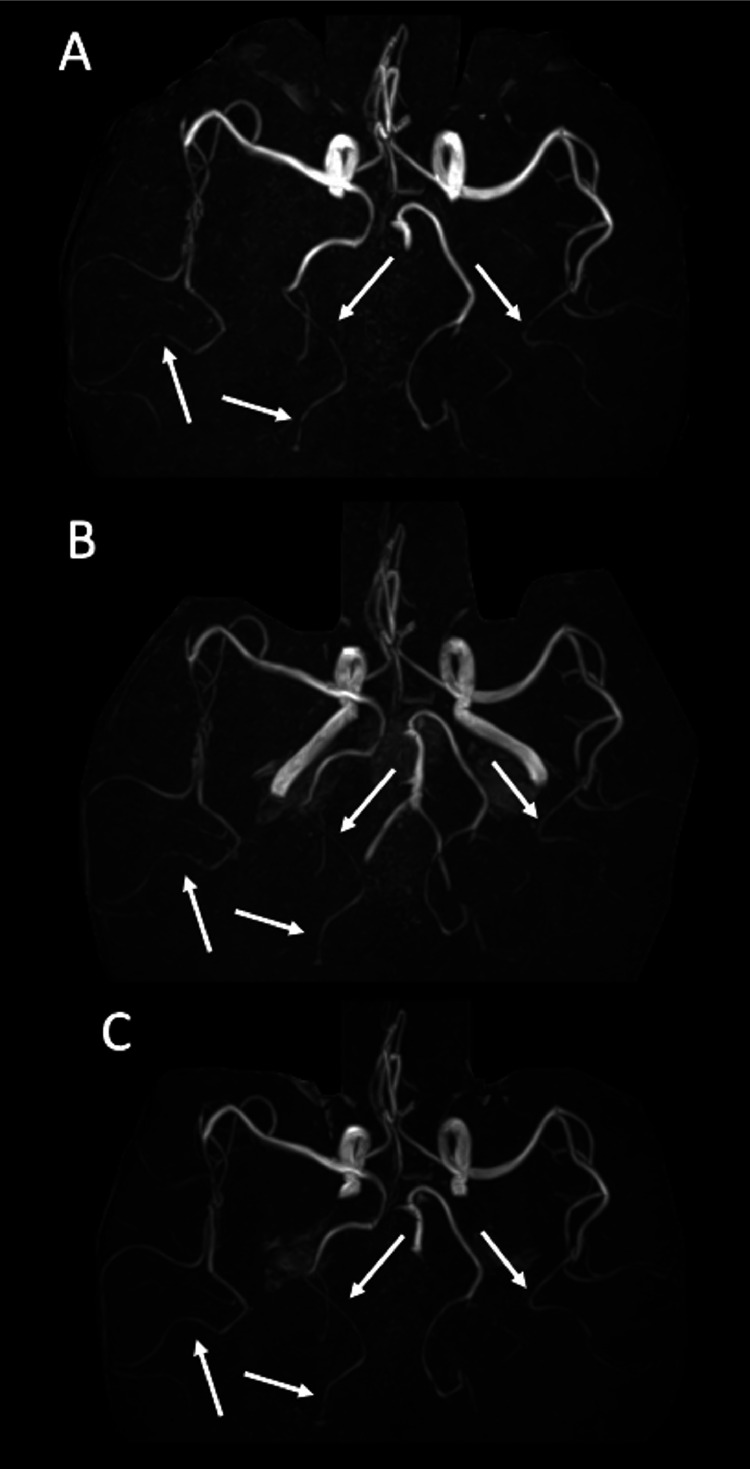
Sequential MRA demonstrating changes in cerebral vasospasm. (A) MRA obtained 3 hours post-onset and (B) 2 days post-onset both reveal scattered vascular stenoses (arrows), including in the right posterior cerebral artery. (C) MRA at 9 days post-onset shows improvement of observed stenoses.

A woman in her sixties with a history of hypercholesterolemia and no prior neurological incidents underwent a dynamic contrast-enhanced abdominal CT scan to investigate an incidentally found splenic artery aneurysm. Iohexol (Omnipaque 300; GE Healthcare, Waukesha, WI, USA), a non-ionic iodine contrast agent, was administered intravenously at the standard dose of 510 mg/kg of body weight and an infusion rate of 2.7 mL/s, which is within the typical range for routine examinations. Immediately following the infusion, she reported a sensation of discomfort in her throat and head area. Within 10 minutes, she exhibited symptoms of anterograde amnesia and disorientation, repeatedly asking “Why am I in the hospital?” and “Have we met before?” An MRI performed 3 hours after symptom onset showed no abnormal signals in the brain parenchyma, but MR angiography (MRA) revealed multiple cerebral arterial stenoses, including the posterior cerebral artery (Figs. 1 and 2). By the following day, her anterograde amnesia had resolved, and she was diagnosed with transient global amnesia (TGA). A repeat MRI 48 hours after symptom onset identified a small high signal area in the right hippocampus on diffusion-weighted imaging (DWI), suggestive of an acute phase micro-infarction. In addition, the cerebral vasospasm showed signs of significant improvement. Another follow-up MRI was performed 9 days after the onset, which showed the hippocampal micro-infarction becoming indistinct, and further improvement of vasospasm on MRA.

## Discussion

We presented a case with transient global amnesia following IV administration of an iodine contrast agent, with a hippocampal micro-infarction considered as a potential cause by transient vasospasm. Amnesia following contrast agent administration is extremely rare, and its exact frequency is unclear. Previous reports have found TGA in 5 out of 4360 cerebral angiography cases, 4 out of 8817 coronary angiography cases, but none in 7659 peripheral angiography [2]. To our knowledge, no case reports have documented amnesia following IV iodine contrast agent administration.

TGA is characterized by acute onset of anterograde and retrograde amnesia that resolves within 24 hours without other neurological symptoms [3]. Its causes remain unclear, with ischemia, seizure, migraine, venous congestion, and psychological factors being considered. The mechanism of TGA is debated, but hippocampal ischemia is one of the leading hypotheses, with imaging studies often showing hippocampal micro-infarctions. Weon et al. reported hippocampal high signals on DWI in 81% of the cases [4]. Bartsch et al. noted that the positivity rate of hippocampal high signals on DWI is highest at 48 to 72 hours after onset and can persist up to 7 to 10 days [5]. In our case, DWI was negative at 3 hours, positive at 48 hours, and negative by day 9, consistent with these findings [4]. It is known that imaging with higher spatial resolution or a high b-value enables earlier identification of hippocampal micro-infarctions. On the other hand, psychological stress has also been suggested as a potential trigger for TGA [6]. The psychological stress associated with undergoing meticulous examinations in a hospital setting may also have played a role in this instance.

MRA conducted 3 hours post-onset in our case revealed multiple vascular constrictions including the right posterior cerebral artery, all of which improved by day 9, indicating transient cerebral vasospasm. It is well known that the hippocampus is vascularized by the upper and lower hippocampal arteries branching from the posterior cerebral artery [7], suggesting that vasospasm of the right posterior cerebral artery or smaller vessels that cannot be captured by MRA could have caused the right hippocampal micro-infarction. No previous reports were found on cerebral vasospasm following IV iodine contrast administration, but one of the serious complications related to iodine-induced vasospasm include Kounis syndrome—a type of acute coronary syndrome with anaphylaxis where histamine and various cytokines and chemokines released by activated mast cells due to contrast hypersensitivity cause coronary artery spasm [8]. The cerebral vasospasm in this case may have occurred by a similar mechanism.

## Conclusion

We encountered a rare case of TGA following administration of iodinated contrast media, with subsequent MRI findings of cerebral vasospasm and hippocampal microinfarction. It is crucial for practitioners conducting contrast-enhanced imaging to recognize that, although rare, contrast agents can lead to cerebral vasospasm and stroke. Careful monitoring and awareness of these potential adverse effects are essential for patient safety.

## Patient consent

Written informed consent for publication of this case report has been obtained from the patient.
